# Thermostable phycocyanin from the red microalga *Cyanidioschyzon merolae*, a new natural blue food colorant

**DOI:** 10.1007/s10811-016-1007-0

**Published:** 2016-11-21

**Authors:** D. Y. Rahman, F. D. Sarian, A. van Wijk, M. Martinez-Garcia, M. J. E. C. van der Maarel

**Affiliations:** 1 Aquatic Biotechnology and Bioproduct Engineering Department, Engineering and Technology Institute Groningen (ENTEG), University of Groningen, Nijenborgh 4, 9747 AG Groningen, The Netherlands; 2 Research Center for Biotechnology, Indonesian Institute of Sciences (LIPI), Jl. Raya Bogor KM 46, 16911 Cibinong, Bogor, Indonesia

**Keywords:** Food colorant, Phycocyanin, Pigments, Red microalga, *Cyanidioschyzon merolae*

## Abstract

The demand for natural food colorants is growing as consumers question the use of artificial colorants more and more. The phycobiliprotein C-phycocyanin of *Arthospira platensis* is used as a natural blue colorant in certain food products. The thermoacidophilic red microalga *Cyanidioschyzon merolae* might provide an alternative source of phycocyanin. *Cyanidioschyzon merolae* belongs to the order Cyanidiophyceae of the phylum Rhodophyta. Its natural habitat are sulfuric hot springs and geysers found near volcanic areas in, e.g., Yellowstone National Park in the USA and in Java, Indonesia. It grows optimally at a pH between 0.5 and 3.0 and at temperatures up to 56 °C. The low pH at which *C*. *merolae* grows minimizes the risk of microbial contamination and could limit production loss. As *C*. *merolae* lacks a cell wall, phycocyanin with a high purity number of 9.9 could be extracted by an osmotic shock using a simple ultrapure water extraction followed by centrifugation. The denaturation midpoint at pH 5 was 83 °C, being considerably higher than the *A*. *platensis* phycocyanin (65 °C). The *C*. *merolae* phycocyanin was relatively stable at pH 4 and 5 up to 80 °C. The high thermostability at slightly acidic pH makes the *C*. *merolae* phycocyanin an interesting alternative to *A*. *platensis* phycocyanin as a natural blue food colorant.

## Introduction

Synthetic dyes are used to provide color to all kinds of food products, confectionary, and beverages (Antello et al. 2008). Consumers have become suspicious about the use of these synthetic colorants as they are linked to having negative effect on children’s behavior (McCann et al. 2007; Arnold et al. 2012). Several large retailers are following major food producers by banning food products containing artificial colorants from their stores. This leads to a growing demand for natural colorants derived from plants and algae. For most of the artificial colorants, natural alternatives are relatively easily available (Wrolstad and Culter 2012). More challenging is to find natural alternatives to the artificial blue colorants such as Patent Blue V (E131) or Brilliant Blue FCF (E133). Blue colors are widespread in nature but it turns out that it is difficult to replicate the blue color. At neutral pH, natural blue colorants are stable but, especially at pH values below 5, they are much less stable and fade quickly (Newsome et al. 2014). Recently the FDA and EFSA have given approval for use of a *Spirulina (Arthrospira) platensis* extract containing high levels of phycocyanin as natural blue food colorant for coloring candy and chewing gum (Code of Federal Regulation 2016). Besides the potential use as food colorant, phycocyanin has also been described as having interesting pharmaceutical and nutraceutical properties (Eriksen 2008).

Phycocyanin is a pigment-protein complex that is part of the phycobilisomes found in cyanobacteria and microalga. Phycobilisomes are large, water-soluble protein complexes attached to the cytoplasmic surface of the thylakoid membrane (Biggins and Bruce 1989) and serve as the major antenna complex harvesting light. Phycobilisomes can make up as much as 20% of the cellular protein content (Glazer 1989). Phycocyanin is an oligomeric protein composed of α and β subunit to which several open chain tetrapyroles are attached (Stec et al. 1999; Padyana et al. 2001; Coyler et al. 2005). The tetrapyrole structures give the typical blue color to phycocyanin while the protein part confers the stability with respect to pH and temperature.

*Arthrospira platensis* phycocyanin has a limited thermostability as it denatures at temperatures above 60 °C (Jespersen et al. 2005; Martelli et al. 2014). In the search for a more thermostable and/or acid stable phycocyanin, the thermoacidophilic red microalga *Cyanidioschyzon merolae* was explored as a source of a blue colorant. *Cyanidioschyzon merolae* is a unicellular microalga belonging to the order Cyanidiophyceae of the phylum Rhodophyta. This species inhabits hot sulfuric springs and geysers in volcanic areas; it grows best at temperatures between 40 and 56 °C and acidic condition of pH 0.5 to 3 (Ciniglia et al. 2004). The phycocyanin of another red microalga, *Galdieria sulphuraria*, has been investigated (Sloth et al. 2006; Sørensen et al. 2012) as this species not only grows at relatively high temperatures and low pH, but also because it can grow heterotrophically in the dark on sugar as well as autotrophically in the light (Gross and Schnarrenberger 1995). *Arthrospira platensis* is grown outdoor in open ponds or raceway systems (Lee 1997; Belay 2013), suffering from productivity loss due to infection as it is difficult to maintain strict hygienic conditions (Richmond and Grobbelaar 1986). The extreme conditions applied to grow red microalga could be advantageous as infections are unlikely at the low pH levels applied. Sloth et al. (2006) and Sørensen et al. (2012) investigated the heterotrophic growth of *G*. *sulphuraria* on glucose in closed fermenters and found a reasonable phycocyanin productivity but concluded that the polysaccharide rich cell wall of *G*. *sulphuraria* makes it difficult to disrupt the cells and extract the phycocyanin. In addition, Sørensen et al. (2012) found that the phycocyanin extracted from *G*. *sulphuraria* cultures was not pure, as a considerable amount of protein and chlorophyll was also extracted. *Cyanidioschyzon merolae* could be a more effective source of phycocyanin as it lacks a cell wall (Lee 2004) making the extraction of the phycocyanin probably more efficient. This paper reports on the production and extraction of phycocyanin from *C*. *merolae* by a simple ultrapure water treatment.

## Materials and methods

### Growth of *C*. *merolae* and phycocyanin extraction

The red unicellular microalga *Cyanidioschyzon merolae* was obtained from National Institute for Environmental Studies (NIES, Japan), catalog no 1332. A stock culture was maintained in Allen medium pH 2 under constant light (100 μmol photons m^−2^ s^−1^) on a shaker at 150 rpm and 40 °C. Allen medium (Allen 1959) consisted of 1.32 g L^−1^ (NH_4_)_2_SO_4_, 0.27 g L^−1^ KH_2_PO_4_, 0.25 g L^−1^ MgSO_4_.7H_2_O, 0.073 g L^−1^ CaCl_2_.2H_2_O, 11 mg L^−1^ FeCl_3_, 2.8 mg L^−1^ H_3_BO_3_, 1.8 mg L^−1^ MnCl_2_, 0.218 mg L^−1^ ZnSO_4_.7H_2_O, 0.05 mg L^−1^ CuSO_4_, 0.023 mg L^−1^ NH_4_VO_3_, and 0.024 mg L^−1^ Na_2_MoO_4_.2H_2_O. The pH of the medium was adjusted to 2.0 with 4 M H_2_SO_4_ and autoclaving at 120 °C for 20 min. A 1-L photo-bioreactor (approximately 100 μmol photons m^−2^ s^−1^ at 40 °C) was used to grow *C*. *merolae* for phycocyanin production. Cultures were harvested by centrifugation at 10,000*×g* for 10 min at 4 °C. The pellet was resuspended in ultrapure water, mixed well for 5 min, and kept for several hours at room temperature. Cell debris was removed by centrifugation at 15,000*×g* at 4 °C for 15 min; the blue colored supernatant was transferred to new tube for further analysis.

### Purification of phycocyanin

To investigate the influence of the time of exposure to ultrapure water on the yield of phycocyanin, 200 mg wet biomass were mixed well with 2-mL ultrapure water (Milli Q purification system) and left at room temperature for up to 300 min. Blue colored supernatant was collected by centrifugation at 15,000*×g* and transferred into new tube. Besides mixing, two other extraction methods were used, bead beating by shaking the suspended cells with a small metal ball at high speed and high-pressure homogenization by means of implosion of the cells.

The crude phycocyanin extract obtained after mixing was further purified by ammonium sulfate precipitation in three steps (20, 40, and 60% saturation). The precipitate was recovered by centrifugation at 10,000*×g* for 30 min, the colorless supernatant was discarded, and the precipitate was dissolved in 50 mM citrate buffer pH 5 at room temperature.

### Determination of concentration

The phycocyanin concentration was estimated by using a spectrophotometer, DR 3900 (Hach-Lange, The Netherlands). Measurement was conducted at 624 and 652, at which phycocyanin and allophycocyanin respectively show maximum absorption (Bennett and Bogorad 1973). The purity of phycocyanin was assessed by calculating the ratio of *A*_624_ to *A*_280_, where *A*_280_ is the absorbance of total protein. The concentration phycocyanin was calculated using the following equation (Silveira et al. 2007):

$$ \mathrm{Phycocyanin}\ \left({\mathrm{mg}\ \mathrm{mL}}^{-1}\right)=\frac{\left({A}_{624}-\left({A}_{652}\times 0.474\right)\right.}{5.34} $$

### Thermostability of phycocyanin

To determine the denaturation midpoint, 1 mL of phycocyanin solution (initial OD *A*_620_ = 0.8) was incubated at pH 5 and different temperatures in a waterbath for 30 min (intervals of 10 °C from 30 to 100 °C). The thermostability of the phycocyanin was measured by incubating samples at pH 5 and 80 °C followed by measuring the absorbance at 624 nm at regular intervals (0–150 min). The remaining concentration of phycocyanin (*C*_R_, %) relative to the initial concentration was calculated using the following equation: *C*_R_ (%) = *C*/*C*_0_ × 100. To determine the pH stability of phycocyanin, the samples were incubated at 80 °C at different pH values from 2 to 5, and the absorbance at 624 nm was measured at regular intervals (0 to 60 min).

## Results and discussion

### Growth of *C*. *merolae* and phycocyanin extraction

**Fig. 1:**
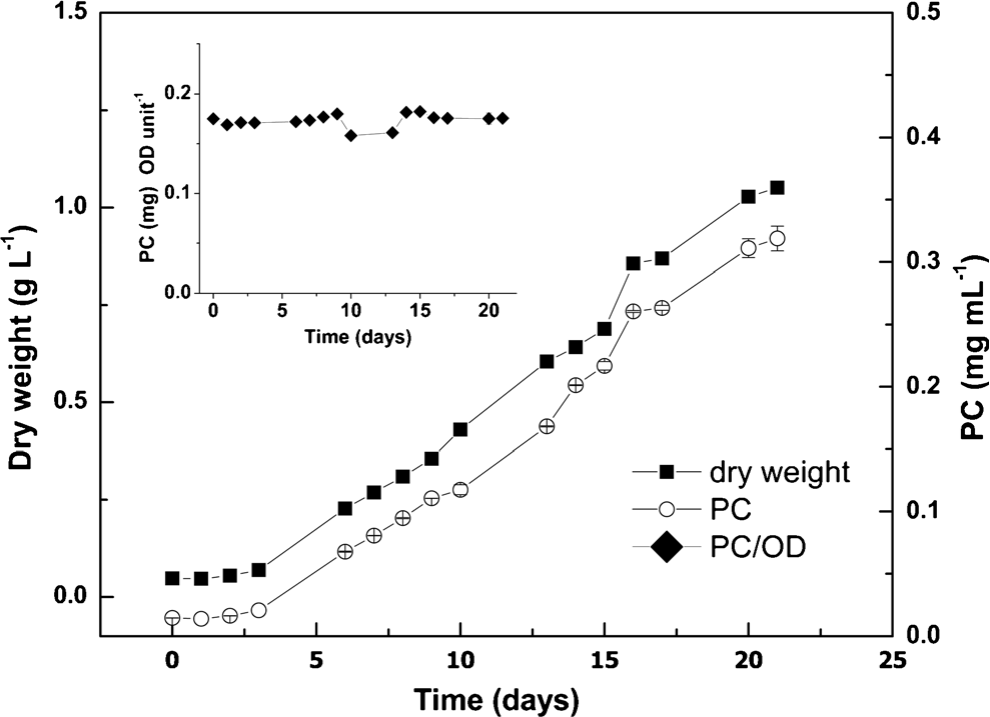
Growth of *C*. *merolae* on air and light and the related phycocyanin production in vivo. (*black squares*), biomass, g dry weight L^−1^; (*white circles*) amount of in vivo phycocyanin measured at 620 nm. In vivo phycocyanin amount per amount of biomass expressed as 1 unit of optical density at 800 nm

**Fig. 2:**
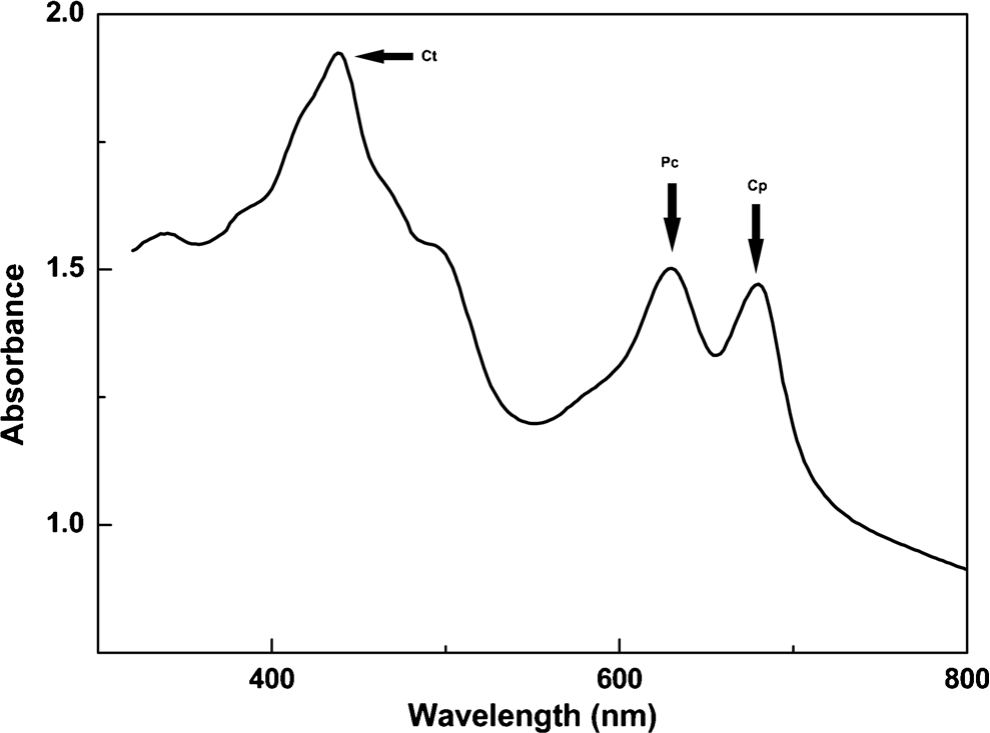
In vivo VIS spectrum (300–800 nm) of *C*. *merolae*. The strong absorption peak at 680 nm is chlorophyll (Cp), the absorption peak around 620 nm corresponds to phycocyanin (Pc). Other absorption peaks in the range of 400–500 nm are from carotenoid (Ct )

The extremophilic red microalga *C*. *merolae* was grown autotrophically in mineral medium at pH 2 and 40 °C with constant illumination (Fig. 1a). After about 3 days, it started to grow with a specific growth rate of 0.15 ± 0.01 day^−1^. At day 21, the cells had reached an OD_800_ of about 1.8, which corresponds to a dry weight of 1.051 ± 0.14 g L^−1^. An in vivo absorption spectrum (300 to 800 nm) was made every day, showing that there were three main absorption maxima, at 430, 620, and 680 nm (Fig. 2). The 430 and 680 nm maxima are typical for chlorophyll (Giltelson et al. 1999), while the 620 nm maximum is typical for phycocyanin (Patel et al. 2005). The amount of phycocyanin per cell did not seem to vary as a constant value of about 17.5 to 18 mg phycocyanin per unit of absorption at 800 nm was found for almost all time points (Fig. 1b).

**Fig. 3:**
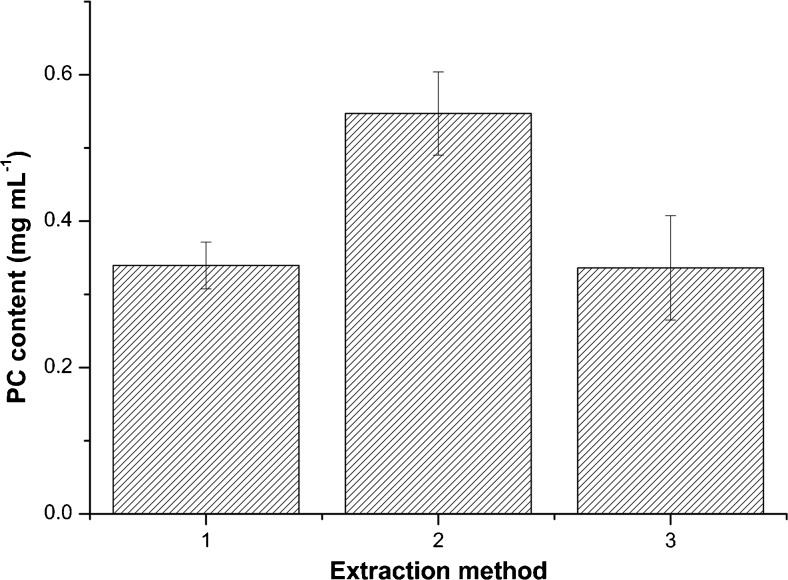
Extraction yield of phycocyanin from *C*. *merolae* with different extraction methods, 1: bead beater, 2: mixing by vortex, and 3: high-pressure homogenizer

To extract phycocyanin from the cells, several cell disruption methods were tested. Bead beating and high-pressure homogenization, both disruptive techniques, were compared with exposure of the cells to an osmotic shock by mixing them with ultrapure water (Fig. 3). The latter method worked best as most phycocyanin (0.55 mg mL^−1^) was found in solution. *Cyanidioschyzon merolae* is known to lack a cell wall (Albertano et al. 2000), making it susceptible to osmotic shocks. When exposed to ultrapure water, the cells take up water and finally lyse, releasing the cellular contents into the water. The water phase mainly contained phycocyanin (*A*_624_, Table 1) with a purity index of 9.92, calculated as the ratio of *A*_624_ to *A*_280_ (protein). Small amounts of chlorophyll were also detected in the water phase (*A*_562_ and *A*_652_, Table 1).

**Table 1:** Absorption at different wavelengths of *C*. *merolae* crude extract and the different fraction of ammonium sulfate treated extract

	Crude extract	Supernatant fraction of 20% (NH_4_)_2_SO_4_	Supernatant fraction of 40% (NH_4_)_2_SO_4_	Pellet fraction of 40% (NH_4_)_2_SO_4_	Supernatant fraction of 60% (NH_4_)_2_SO_4_	Pellet fraction of 60% (NH_4_)_2_SO_4_
Volume of sample (mL)	40	40	40	10	40	10
Dilution	1:5	1:5	1:5	1:20	1:5	1:20
A_652_	0.098	0.096	0.032	0.059	0.019	0.031
A_624_	0.496	0.469	0.167	0.271	0.042	0.147
A_562_	0.145	0.135	0.049	0.135	0.017	0.048
A_280_	0.05	0.051	0.025	0.015	0.073	0.014
Purity index	9.92	9.26	6.78	18.07	0.58	10.78
Total C-PC	16.8	15.8	5.6	9.2	1.2	5
Yield	100	94.31	33.81	54.06	7.38	31.32

### *C*. *merolae* phycocyanin extracted by osmotic shock has a high purity number

**Fig. 4:**
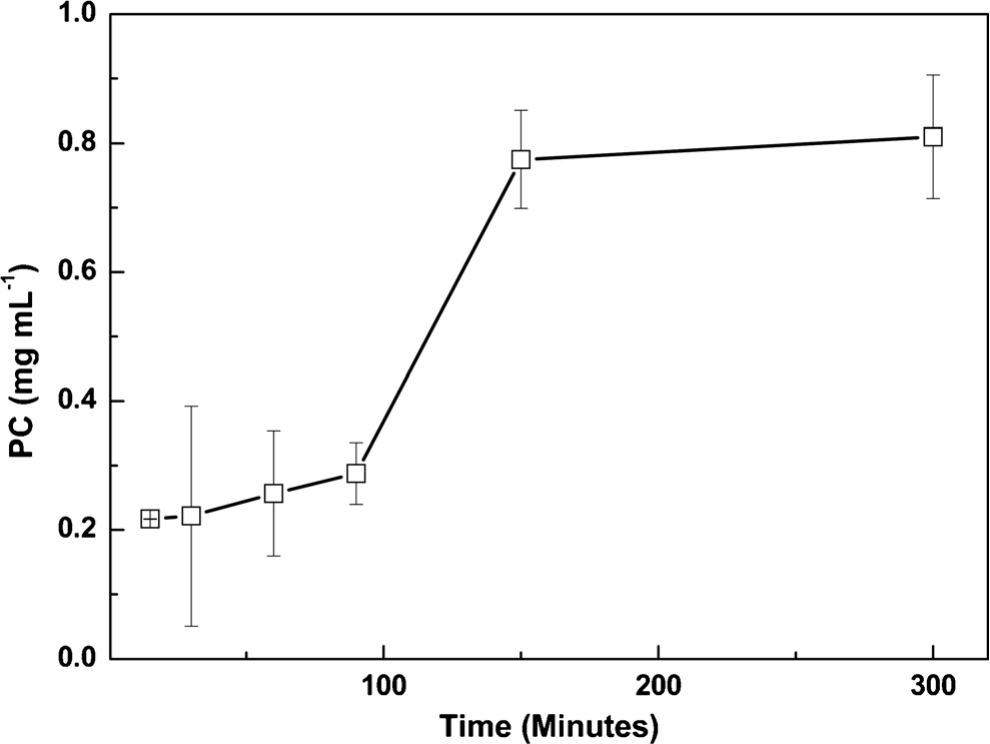
The effect of exposing *C*. *merolae* cells to ultra pure water for an increasing amount of time (in min)

The duration of the osmotic shock had a clear effect on the amount of phycocyanin extracted (Fig. 4). Incubating *C*. *merolae* cells in ultrapure water for up to 100 min gave relatively low amounts of phycocyanin in the water phase (up to 0.28 mg mL^−1^). However, a steep increase in the amount of phycocyanin was observed between 100 and 150 min incubation; after 150 min, almost 0.81 ± 0.09 mg mL^−1^ phycocyanin was released. Longer incubation did not result in a significant increase in the amount of phycocyanin released. The purity of the phycocyanin extract obtained with ultrapure water treatment is considerably higher than that of extracts obtained from *Spirulina*, with a purity index ranging from 0.46 to 2.78 (Silveira et al. 2007; Liao et al. 2011), from *Synechococcus* with a purity index of 2.2 (Gupta and Sainis 2010), and from the closely related red microalga *G*. *sulphuraria* of only 1 (Sørensen et al. 2012). The reason for the much higher purity index found for the phycocyanin extracted from *C*. *merolae* compared to other phototrophs is that the latter have a (thick) cell wall requiring a mechanical treatment to disrupt the cells and release the phycocyanin. As a result of the mechanical treatment of *G*. *sulphuraria*, also chlorophyll *a* and carotenoids are released into solution (Sloth et al. 2006), resulting in much lower purity number for phycocyanin. Applying different purification techniques such as aqueous two phase extraction with polyethylene glycol and water (Rito-Palomares et al. 2004), ammonium sulfate precipitation of expended bed absorption in combination with chromatography resulted in higher purity numbers (Zhang and Chen 1999; Niu et al. 2007; Yan et al. 2011), but these are still considerably lower than the 9.9 found for *C*. *merolae*. Phycocyanin solution with a purity index of at least 0.7 are considered to be a food grade, while a purity index of at least 4 is considered to be analytical grade (Cisneros and Rito-Palomares 2004). The phycocyanin extracted from *C*. *merolae* grown cells has a high purity index any further purification is not required to use it as analytical grade material.

**Fig. 5:**
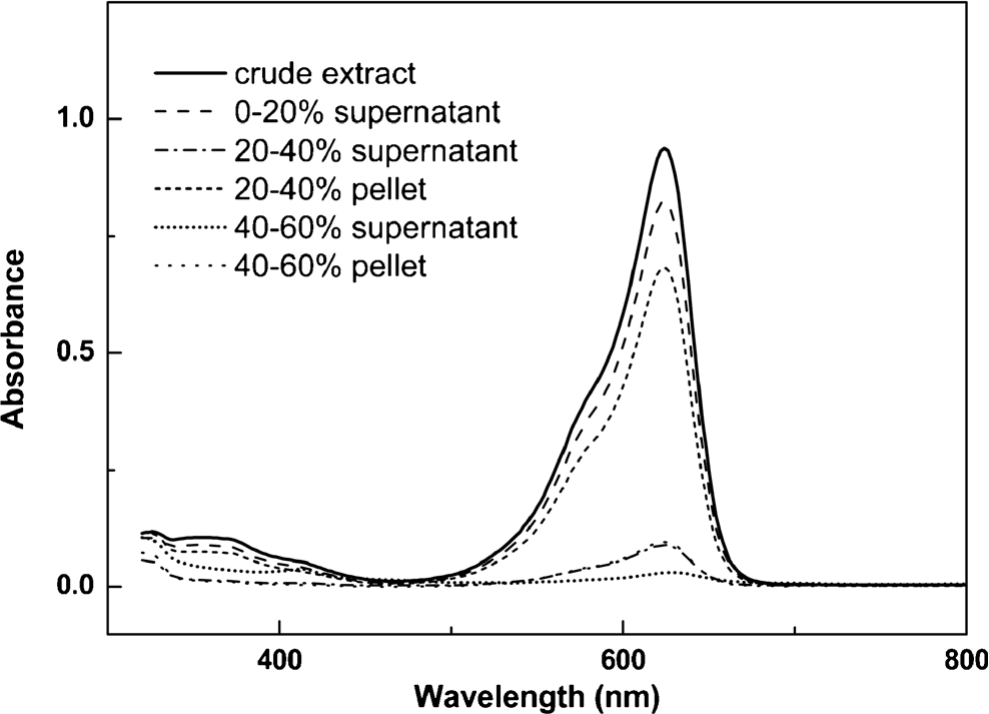
Visible absorption spectra of soluble and precipitated phycocyanin from *C*. *merolae* at different ammonium sulfate concentrations

The ultrapure water extract containing phycocyanin was further purified by ammonium sulfate precipitation at 20–40% saturation resulting in a concentrated phycocyanin solution (54% yield) with a purity index of 18.07 (Table 1). The pellet fraction 20–40% ammonium sulfate was dissolved in citrate buffer (pH 5) and was used to further characterize the *C*. *merolae* phycocyanin. The absorption spectrum of this fraction showed a clear *λ*_max_ at 624 nm (Fig. 5), being the phycocyanin, and shoulder at 562 nm, indicative for phycoerythrin (Cisneros and Rito-Palomares 2004). The *A*. *platensis* phycocyanin has a *λ*_max_ of 616 nm at pH 5 and 620 at pH 7 (Jespersen et al. 2005). The thermophile *Synechococcus lividus* phycocyanin has *λ*_max_ of 609 nm at pH 6 (Edwards et al. 1997), while *Phormidium luridum* phycocyanin has a *λ*_max_ at pH 6 of 622 nm (Edwards et al. 1996). Glazer and Fang (1996) even reported a *λ*_max_ of about 650 nm at pH 3 for the phycocyanin of *Synechococcus* sp.

### *C*. *merolae* phycocyanin is thermostable

**Fig. 6:**
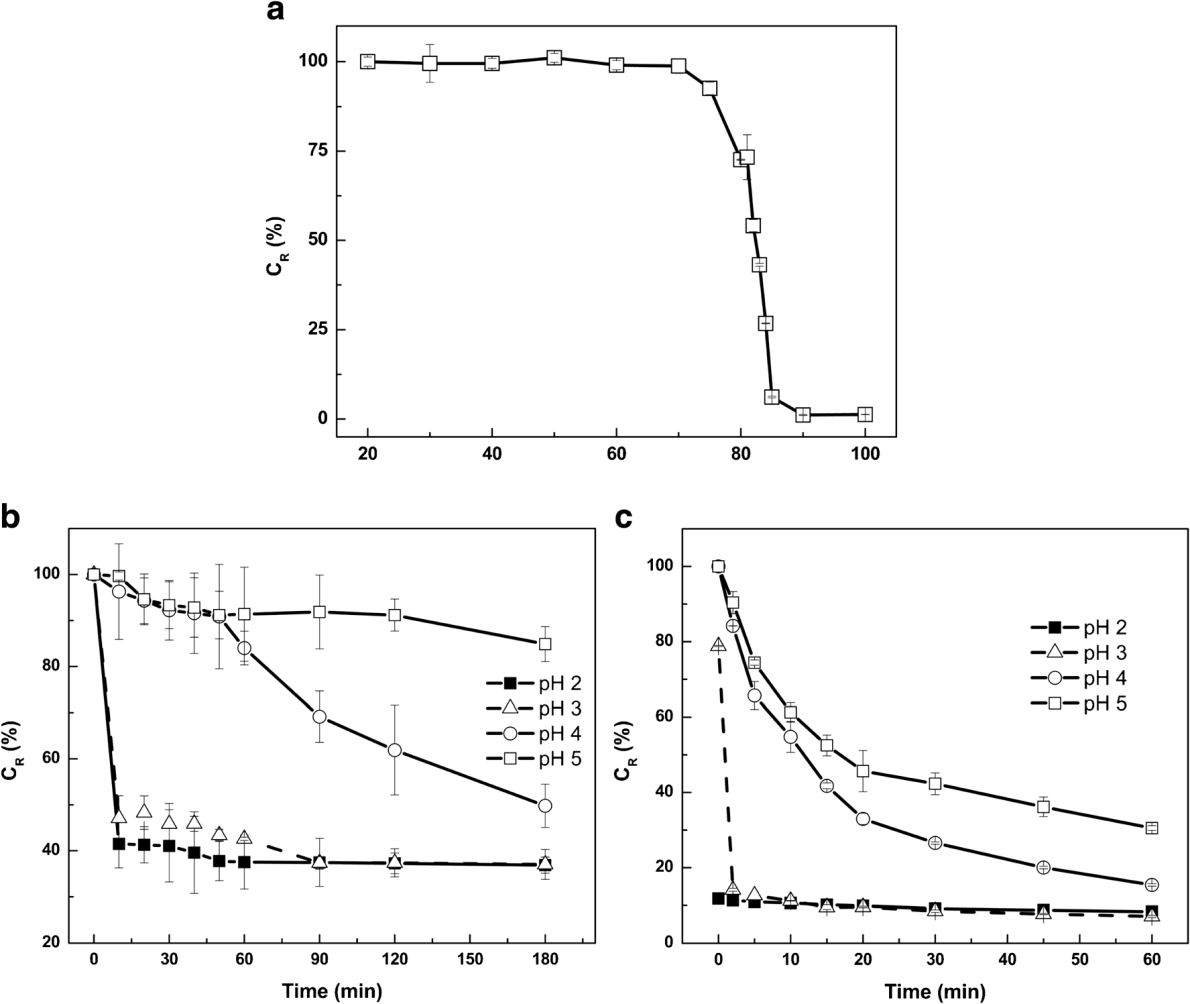
Characteristics of phycocyanin from *C*. *merolae*. **a** Effect of increasing temperatures on the solubility (*C*_R_ (%)) of phycocyanin (incubation time: 30 min). **b** pH stability over a period of 180 min at 27 °C and **c** at 80 °C. *Black squares* pH 2, *Triangles* pH 3, *white circles* pH 4, *White squares* pH 5

The alpha and beta subunit of the phycocyanin having a molecular weight of 17–18 kDa were clearly visible on an SDS-polyacrylamide gel (data not shown). This is in agreement with the molecular weight of other phycocyanin reported so far (Glazer and Fang 1996; Chaiklahan et al. 2011). As *C*. *merolae* can grow at relatively high temperatures up to 55 °C, it is to be expected that the phycocyanin is relatively thermostable. The purified phycocyanin was incubated for 30 min in citrate buffer (pH 5) at temperatures varying from 20 to 100 °C (Fig. 6a). Up to 75 °C, the phycocyanin was soluble and remained clearly blue; at temperatures above 75 °C, it started to precipitate and at 90 °C, it had completely precipitated. The denaturation midpoint, which can be defined as that temperature (*T*_m_) at which 50% of the phycocyanin is still in solution (*C*_R_ = 50%), of *C*. *merolae* phycocyanin is at 83 °C (Fig. 6a). At room temperature and pH 5, the 40% ammonium sulfate purified phycocyanin fraction was stable; more than 85% stayed in solution for 180 min (Fig. 6b). At pH 4, this fraction was already less stable; about 50% of the phycocyanin was lost after 180 min of incubation (Fig. 6b). At 80 °C and pH of 4 or 5, the color faded much more rapidly (Fig. 6c). At pH of 2 and 3, the blue color disappeared within several minutes both at room temperature and at 80 °C (Fig. 6b, c). The half-life of the 40% ammonium sulfate purified phycocyanin at room temperature was several hundred minutes at pH 4 and 5 while at pH 2 and 3, the phycocyanin faded to colorless in less than 5 min (Table 2). At high temperature of 80 °C, the phycocyanin had a half-life of 29 to 40 min at pH 4 and 5, respectively. At pH 2 and 3 and 80 °C, the phycocyanin lost its color within several minutes (Table 2).

**Table 2:** Half-life of ammonium sulfate (40% saturation) purified phycocyanin of *C merolae* at room temperature (22 °C) and 80 °C at pH 3, 4 and 5

Temperature (°C)	pH	Half-life (min)
22	3	<5
	4	>400
	5	>1200
80	3	<5
	4	29
	5	40

Phycocyanin predominantly exists as a hexamer at pH 5, and it is believed that the hexameric form gives some protection against denaturation (Edwards et al. 1996). At pH 7, it is predominantly in a monomeric or trimeric form, resulting in lower thermostability (Edwards et al. 1997; Jespersen et al. 2005). Very likely, the *C*. *merolae* phycocyanin is also present in monomeric or trimeric form at low pH, as it looses its thermostability rapidly at these low pH values. The stability of the *C*. *merolae* phycocyanin at higher temperatures and a pH of 4 or 5 is much better than that of the *S*. *platensis* phycocyanin. The *T*_m_ of the *A*. *platensis* phycocyanin is between 55 and 62 °C (Jespersen et al. 2005; Martelli et al. 2014). The *C*. *merolae* phycocyanin protein sequence contains four cysteine residues, whereas the *A*. *platensis* phycocyanin sequence, being 75% identical to the *C*. *merolae* sequence, has two cysteine residues (UniProtKB-P72509). Cysteine can form covalent disulfide bonds that contribute to the thermostability of a protein (Fass 2012). Adding high amounts of sugars (40 to 55%) such as fructose or glucose improved the thermostability of *A*. *platensis* phycocyanin, indicating that it could be used in high sugar food products such as confectionary and pastries (Martelli et al. 2014). As *C*. *merolae* phycocyanin already has a higher thermostability of its own, it could be used in low sugar products that are exposed to higher temperatures during production.

## Conclusions

Phycocyanin can easily be extracted from autotrophically grown *C*. *merolae* cells by an osmotic shock procedure with ultrapure water. The phycocyanin obtained in this way has a high purity number (9.9) and is thermostable up to 83 °C at neutral and slight acidic pH. These properties make the *C*. *merolae* phycocyanin an interesting alternative to *A*. *platensis* phycocyanin as a natural blue food colorant.
